# Pregnancy and COVID-19

**DOI:** 10.3390/jcm11226645

**Published:** 2022-11-09

**Authors:** Thomas Ntounis, Ioannis Prokopakis, Antonios Koutras, Zacharias Fasoulakis, Savia Pittokopitou, Asimina Valsamaki, Athanasios Chionis, Evangelia Kontogeorgi, Vasiliki Lampraki, Andria Peraki, Athina A. Samara, Sevasti-Effraimia Krouskou, Konstantinos Nikolettos, Panagiotis Papamichalis, Alexandros Psarris, Vasilios Pergialiotis, Marianna Theodora, Panos Antsaklis, Alexandros Daponte, Georgios Daskalakis, Emmanuel N. Kontomanolis

**Affiliations:** 1 1st Department of Obstetrics and Gynecology, National and Kapodistrian University of Athens, General Hospital of Athens ‘ALEXANDRA’, Lourou and Vasilissis Sofias Ave, 11528 Athens, Greece; 2 Department of Internal Medicine, Koutlimbaneio and Triantafylleio General Hospital of Larissa, Tsakalof Str. 1, 41221 Larisa, Greece; 3 Department of Obstetrics and Gynecology, Laikon General Hospital of Athens, Agiou Thoma Str. 17, 11527 Athens, Greece; 4 Department of Embryology, University General Hospital of Larissa, Mezourlo, 41110 Larissa, Greece; 5 Department of Obstetrics and Gynecology, Democritus University of Thrace, 6th km Alexandroupolis-Makris, 68100 Alexandroupolis, Greece; 6 Intensive Care Unit, General Hospital of Larissa, 41110 Larissa, Greece; 7 Department of Obstetrics and Gynecology, University General Hospital of Larissa, Mezourlo, 41110 Larissa, Greece

**Keywords:** COVID-19, SARS-CoV-2, pregnancy, maternal outcomes, obstetric outcomes, neonatal outcomes, vaccination

## Abstract

Evidence indicates that SARS-CoV-2 infection increases the likelihood of adverse pregnancy outcomes. Modifications in the circulatory, pulmonary, hormonal, and immunological pathways induced by pregnancy render pregnant women as a high-risk group. A growing body of research shows that SARS-CoV-2 infection during pregnancy is connected to a number of maternal complications, including pneumonia and intensive care unit (ICU) hospitalization. Miscarriages, stillbirth, preterm labor, as well as pre-eclampsia and intrauterine growth restriction are also among the most often documented fetal implications, particularly among expecting women who have significant COVID-19 symptoms, often affecting the timing and route of delivery. Thus, prevention of infection and pharmacological treatment options should aim to minimize the aforementioned risks and ameliorate maternal, obstetric and fetal/neonatal outcomes.

## 1. Introduction

On 11 March 2020, the World Health Organization proclaimed the coronavirus disease 2019 (COVID-19) to be a pandemic. [1] It is induced by the severe acute respiratory syndrome coronavirus 2 (SARS-CoV-2), an RNA betacoronavirus that infects humans via angiotensin-converting enzyme 2 (ACE2), a receptor on the membrane of epithelial cells. This receptor is most commonly found in type II alveolar cells of the lungs and in the mucosa of the oral cavity, affecting both upper and lower respiratory systems [2,3]. It can also be present in organs such as the heart or the intestine, which can be additionally infected through systemic circulation [4]. Respiratory secretions are considered to be the main route of person-to-person transmission of SARS-CoV-2. Direct contact of an infected individual’s virus carrying droplets, originating from speaking, sneezing, or coughing, with the mucous membranes of someone nearby can result in an infection. SARS-CoV-2 is expected to have up to a 14-day incubation period, with most cases occurring about 5 days after exposure [5]. The current review aims to summarize the available data and research on COVID-19 in pregnancy, assessing the effects of SARS-CoV-2 infection on pregnant women, particularly on maternal, obstetric, and neonatal outcomes.

## 2. Materials and Methods

For this review, the authors searched MEDLINE (National Library of Medicine, Bethesda, Maryland, MD, USA; January 1980 to July 2020) and the Cochrane Register of Controlled Trials (The Cochrane Collaboration, Oxford, UK). An electronic search approach included the phrases ‘SARS-CoV-2’, ‘severe acute respiratory syndrome’, ‘Coronavirus disease’, ‘COVID-19’, and ‘pregnancy’. To find further research of interest, the references of the selected publications and review articles were evaluated. To select possibly relevant papers for this study, the authors evaluated all of the citations returned from the computerized search.

## 3. COVID-19 in Pregnancy

Due to the cardiovascular, pulmonary, hormonal, and immunological changes that accompany pregnancy, pregnant women are believed to be at a heightened risk during the pandemic [2,3,4,6,7,8,9,10]. More specifically, the hormonal fluctuations and the prevalence of a Th2 cell-mediated immunological environment increase pregnant women’s susceptibility to infections, while the elevated maternal oxygen needs, together with the diminished capacity of the lungs, due to the upraised level of the diaphragm, reduce women’s tolerance to hypoxia and dyspnea. Thus, pregnant women’s infection with SARS-CoV-2 has been correlated with more severe morbidity, affecting both the mother and the fetus [11,12,13,14,15]. 

SARS-CoV-2 infects respiratory epithelial cells, triggering an immunological response defined by the production of pro-inflammatory cytokines and a modest interferon response. A downstream signaling pathway due to membrane-bound immune receptors activates the Th1 cells’ and CD14+ CD16+ monocytes’ proinflammatory response. The subsequent infiltration of macrophages and neutrophils into lung tissue initiates a cytokine storm. This cytokine storm in COVID-19 is characterized by elevated levels of IL-6 and TNF- expression. A possible mechanism for this storm has been proposed by Hirano and Murakami, through the angiotensin 2 (Ang-II) pathway. Particularly, SARS-CoV-2 reduces the ACE2 expression and increases the expression of Ang-II. This stimulates the production of TNF-α and the soluble form of IL-6Ra via disintegrin and metalloprotease 17. IL-6 with its receptor forms a complex which activates the transcription 3 (STAT3). STAT3 and NF-B activation by SARS-COV-2 induce the production of proinflammatory cytokines and chemokines, particularly endothelial growth factor, monocyte chemoattractant protein 0 (MCP-1) and interleukin 8 (IL8). The levels of IL-1, IL-2, IL-6, IL-7, IL-10, IP-10, MCP-1, TNF-, macrophage inflammatory protein 1 alpha, and granulocyte-CSF have been shown to be elevated in individuals with a more severe condition, according to previous research. Among them, IL-6 presumably is the major cause of severe lung inflammation and pulmonary function. In addition, Liu et al. discovered a drop in lymphocyte counts, particularly CD8+T cells, and a rise in neutrophil counts in these patients. The severity of the disease is related to T-cell lymphopenia and the dynamic cytokine storm and especially the latter is considered as an important cause of death in these patients. A recent meta-analysis revealed that pregnant women had a crucially increased risk for severe COVID-19 characterized by this cytokine storm [16,17,18].

Nonimmunization; non-Caucasian ethnicity; a body mass index above 25 kg/m^2^; a pre-pregnancy co-morbidity such as diabetes or hypertension; a maternal age of 35 years or older; increased socioeconomic deprivation; and employment in healthcare or other public-facing occupations are risk factors for both infection and hospitalization with COVID-19 [19,20]. In a study of nearly 400,000 women of reproductive age with symptomatic COVID-19, the Centers for Disease Control and Prevention (CDC) found that pregnant women had a vastly greater adjusted risk ratio (aRR) for mortality and morbidity than non-pregnant women of reproductive age (aRR 5 1.7, 95% CI 1.2–2.4) [21]. Comparable to this, Rozo et al. showed that the risk of mortality for pregnant women was considerably higher than that for non-pregnant women of reproductive age (aRR 5 1.82, 95% CI) [22].

Over two-thirds of confirmed pregnant women do not exhibit any symptoms, and pregnant women do not appear to be more or less susceptible to contracting SARS-CoV-2 than the general population [20] In symptomatic COVID-19 pregnant women, fever, coughing, shortness of breath, headaches, exhaustion, myalgia or arthralgia and taste loss appeared to be some of the most common symptoms. Fever, usually with a median temperature between 38.1 and 39 °C, is described as the most prevalent symptom and appears in the majority of the patients [13,14,15,23,24,25,26]. The aforementioned symptoms follow two different roads. Either they improve with early identification and conservative treatment or they lead to dyspnea and productive cough. Various cohorts have demonstrated that the median time of the appearance of dyspnea is 6 days after exposure. Secondary infection is always possible in about 12 days, while viral shedding is completed in about 20 days. Zhou et al. reported that the median time from the beginning of the illness until discharge was 22 days and the median time for death was 185 days. Fever stayed for at least 12 days and cough for 19 days in people who survived [26]. Concerning admission to the hospital, development of ARDS (acute respiratory distress syndrome) and admission to ICU with the need for mechanical ventilation is 8, 8.2, and 10 days, respectively. 

Elevated C-reactive protein and a decreased lymphocyte count were the most prevalent laboratory results in COVID-19-positive pregnant women, but leukocytosis was seldom reported. Physiologically, a mild increase in leukocytes occurs in the third trimester; thus, Liu et al. showed that the leukocytosis in COVID-19 pregnant patients was within the high normal range. In addition, procalcitonin and IL-6 levels were higher in pregnant women compared to non-pregnant. An exception was noticed in the ferritin serum levels which were not significantly higher, probably as a result of frequent iron-deficiency-related anemia during pregnancy. Previous data suggest that there is an additional risk of venous thromboembolism in infected pregnant patients. A retrospective comparative study of 2021 showed significantly lower NT-proBNP and troponin levels in pregnant patients. Available data suggest that NT-proBNP constitutes an independent risk factor for ICU admission, need for mechanical ventilation, coagulopathy, and in-hospital death in cases of severe COVID-19 disease [9]. 

RT-PCR of nasopharyngeal or oropharyngeal samples is the diagnostic gold standard for COVID-19 infection. RT-PCR involves the reverse transcription of RNA into complementary DNA and the proliferation of specific DNA targets. qRT-PCR is a more precise molecular-based test that can detect and quantify a limited number of antibodies rapidly. New techniques have been developed which are more accurate and easier to perform, in the form of kits such as RT-LAMP and CRISPR-Cas12. The rapid antigen tests (RAD) detect viral antigen by the immobilized coated SARS-CoV-2 antibody on the device within 30 min, without the need for a specialized instrument. However, RAD tests have been associated with more false-negative results. X-rays constitute an important tool in diagnosing pneumonia and assessing its severity. Ai et al. found that the majority of patients had initial positive chest CT scans before or within six days of the first positive RT-PCR test, suggesting that a CT scan can aid in the early diagnosis of the infection. The most characteristic CT features in infected patients are multiple peripheral ground-glass opacities (GGO), consolidation, and interlobular septal thickening. When it comes to pregnancy, patients may be concerned about radiation. However, imaging should be performed when there is a high suspicion of COVID-19 or when is needed to determine the treatment, since a delay in recognition of the severity of the disease increases maternal mortality [27,28,29].

The course of infection during pregnancy has changed due to different SARS-CoV-2 variants over time. There is mounting evidence that, especially during the third trimester, pregnant women may be more susceptible to serious COVID-19 than non-pregnant women. A study in Scotland showed that hospital admission was needed in 7% of infections in the first trimester, 11% and 34% in the second and third trimesters, respectively [30]. A UK study showed that during the periods of Alpha and Delta variants’ predominance, more severe maternal disease and worse pregnancy outcome were reported compared to the wildtype period. Consequently, as the Delta variant was dominant, an increase in the number of pregnant patients with severe-to-critical illness was noticed. Notably, one in ten symptomatic women admitted to hospital with the Alpha variant required intensive care, compared to one in seven with the Delta variant, indicating that the Delta variant may be associated with a more severe condition. Moreover, although the Omicron variant is more contagious than the Delta variant, it is associated with less severe disease; however, it is still linked to unfavorable maternal and newborn outcomes, especially in uninfected pregnant women [20]. Eid et al. showed that vaccinated pregnant patients had milder symptoms and considerably fewer hospital or ICU admissions than unvaccinated patients. Furthermore, in unvaccinated pregnant patients, the use of monoclonal antibodies provided a significant delay in the disease progression, while in vaccinated patients, there was no additional benefit. The protective effect of vaccination seems to extend to the fetus and newborn, leading to a decreased rate of COVID-19 infection during the first 6 months of life [30].

### 3.1. COVID-19 and Maternal Complications

As indicated, COVID-19 is a very serious infectious disease, especially in pregnant women, who are considered a high-risk group, as there is always the possibility of developing all the aforementioned symptoms, along with complications. ARDS (acute respiratory distress syndrome) is the most common and severe complication, followed by sepsis and septic shock, kidney acute injury, and cardiac acute injury [26], Additionally, severe pneumonia was also present in a significant number of pregnant patients which, in the majority of the studies, leads to the observation that pregnancy can indeed amplify the risk of a SARS-CoV-2 infection to develop into pneumonia [31]. Furthermore, pregnant women have a stronger chance of being admitted to an intensive care unit, in contrast to pregnant women without the disease. Giampiero Capobianco et al. mentioned that 13% of pregnant women were admitted to the ICU [4]. Similarly, Ioannis Bellos et al. stated that 11% of infected pregnant women evolved into a dismal outcome and had to attend the ICU, while Kuma Diriba et al. inferred that 28% of the cases were transferred to an ICU. As a result, if the mother’s conditions worsen, even the danger of death lurks [5]. Cases of maternal deaths have been reported in the bibliography. Karami et al. described the case of a pregnant woman who was admitted to the hospital with COVID-19. Soon after admission, her temperature rose up to 40 °C and she appeared to have metabolic alkalosis, and later on, acute respiratory distress syndrome (ARDS). She spontaneously delivered a dead cyanotic neonate and the next day she developed multiorgan failure and died [12]. Zamaniyan et al. revealed the case of a woman with dismal pneumonia symptoms, who delivered urgently with cesarean section and soon after, she developed acute respiratory distress syndrome and peritoneal dialysis that deteriorated and led to her death [32]. Other maternal deaths have also been reported in several studies [25,33,34]. Therefore, even though dismal outcomes do not represent the majority of the cases, significant incidents have been showcased and cannot be left out of consideration, as they can be determinant for both mother and her fetus. 

Other studies also reported several other pregnancy complications such as pre-eclampsia, gestational diabetes, hypertensive disorders, hypothyroidism, and anemia but no certain conclusion in coordination to COVID-19 could be reached [1,3,4,6,7,8,9]. J. Juan et al. observed that these complications did not appear with a higher frequency in pregnant women with COVID-19 than in those without [4]. However, Daniele Di Mascio et al. and Kuma Diriba et al. mentioned a higher tendency of pre-eclampsia in infected pregnant women.

Pre-eclampsia (PE) and COVID-19 are both multisystematic disorders that have a variety of manifestations. They share overlapping pathogenic pathways and their symptoms reflect extensive endothelial dysfunction (ED), which frequently leads to vasoconstriction and end-organ ischemia. Pre-eclampsia and COVID-19 are symptoms of ED brought on by elevated levels of the anti-angiogenic hormone angiotensin II (Ang-II) and the circulating anti-angiogenic molecules sFlt1. Both of these disorders start in the placenta and lungs, respectively, and they both finish in the endothelium. During pregnancy, severe COVID-19 can cause PE-like symptoms. Furthermore, COVID-19 at pregnancy is independently related to PE, according to a new sub-analysis from the INTERCOVID research sample. According to evidence, PE is brought on by an imbalance of soluble plasmatic anti- and pro-angiogenic factors, which are essential for maintaining the vascular endothelium. Women with PE have lower levels of placental growth factor (PlGF), a potent angiogenic factor, and higher levels of soluble FMS-like tyrosine kinase 1 (sFlt-1), the primary anti-angiogenic factor, before clinical manifestation. Moreover, findings show that excessively high sFlt-1/PlGF ratios may aid in the detection of placental dysfunction even in SARS-CoV-2-positive pregnant women. Both COVID-19 and PE are connected to hypocalcemia, elevated lactate dehydrogenase and sFlt1, hypoalbuminemia, elevated levels of IL-6 and D-dimer, thrombocytopenia, and proteinuria [35,36].

### 3.2. COVID-19 and Fetal Complications

SARS-CoV-2 infection during pregnancy can also affect the fetus directly. Firstly, cases of miscarriages and perinatal deaths have been stated. An approximately twofold increase in the chance of stillbirth and a possible rise in the prevalence of small-for-gestational-age infants are both linked to maternal COVID-19 infection. Preterm births that are largely iatrogenic appear to be two to three times as common than background births in women with symptomatic COVID-19 [20]. Pradip Dashraath et al. outlined that approximately 2% of infected pregnancies have resulted in a miscarriage [2]. In Jie Yan’s study, an unexpected miscarriage occurred in a 5-week pregnancy, accompanied by fever and fatigue [15]. Additionally, J. Juan et al. mentioned four cases of miscarriages [4]. In the same study, a neonatal death due to asphyxia was inferred. One case of neonatal death was also described by Zhu H et al. [34]. The newborn appeared with gastric bleeding, refractory shock, disseminated intravascular coagulation, and multiple organ failure, possibly caused by viremia, combined with an immature immune system. Moreover, Ioannis Bellos et al. showcased three incidents of stillbirth and two of neonatal deaths, both delivered by seriously infected women who were transmitted to the ICU [10]. Daniele Di Mascio et al. revealed in their study that in 7% of the infected pregnancies, perinatal death occurred. To be more exact, there was one case of stillbirth and one of neonatal death [35]. Some stillbirths were also mentioned by John Allotey et al. [6]. Both studies underlined the higher frequency of miscarriages and perinatal deaths among infected pregnant women with SARS-CoV-2 than among women without the disease. In India, the stillbirth incidence during the COVID-19 pandemic appeared notably higher than what was documented in 2019 (13.9 per 1000 births) [8]. The Netherlands Obstetric Surveillance System’s most recent statistics show that 58 of the 9620 known SARS-CoV-2-infected pregnancies in The Netherlands ended in stillbirth from 1 March 2020 to 7 December 2021. Nevertheless, there was no information provided for the same time about the number of stillbirths in uninfected pregnancies [11]. Studies with a significant study population have shown that SARS-CoV-2-positive women have a higher incidence of stillbirth than women who did not contract the virus [37,38,39,40]. However, data are debatable, since some smaller studies found no appreciable increase in the stillbirth incidence among SARS-CoV-2-positive pregnant women. It is vital to acknowledge whether any rise in the stillbirth rate is brought on directly by the maternal SARS-CoV-2 infection or due to modifications in healthcare availability, in pregnant women’s or medical professionals’ behavior during the pandemic.

The most common fetal complication of COVID-19 appears to be preterm birth [9]. Giampiero Capobianco’s review mentioned that preterm births appeared in almost all of the studies, with a mean percentage of 23% among the cases [7]. Several other studies confirmed the rate of preterm births among pregnant women infected with SARS-CoV-2 to be between the rank of 25–44% [26,32,33,34,35,36]. Moreover, Pedro Castro et al. reported that 19.41% of the infected pregnancies were delivered before 37 weeks and 15% before 34 weeks [33]. Additionally, Daniele Di Mascio et al. stated that preterm birth below 37 and 34 gestational weeks occurred in 41.1% and 15% of the pregnancies, respectively [3]. The same study underlined the higher tendency of preterm birth among pregnant women with COVID-19, in comparison to those without the disease. In agreement with the above statement were John Allotey et al., Rong Yang et al. and Chiu-Lin Wang et al. [6,11,24]. 

In general, COVID-19-diagnosed women had lower rates of spontaneous labor onset but greater rates of caesarean section, indicating the higher rates of obstetric morbidity in this group. The INTERCOVID Multinational Cohort Study revealed that the increased risk in this group (RR, 1.97; 95% CI, 1.56–2.51) is attributable to the fact that 83% of preterm deliveries (*n* = 130) among women with a COVID-19 diagnosis had medical evidence, with pre-eclampsia/eclampsia/HELLP (31 [24.7%]), small for gestational age or intrauterine growth restriction (24 [15.5%]) [41].

As mentioned above, other fetal complications possibly correlated to SARS-CoV-2 infection were observed to be SGA or IUGR and pre-eclampsia. Pradip Dashraath et al. stated that fetal growth restriction occurred in approximately 10% of the pregnancies infected with SARS-CoV-2. This phenomenon was caused by the lack of oxygenation of the fetus, mainly due to the prolonged respiratory compromise and the hypoxia of the mother [2]. Similarly, Daniele Di Mascio et al. stated that 43% of the fetuses presented fetal distress, while Kuma Diriba et al. reported that SARS-CoV-2 increased the risk of fetal distress among pregnant women [42,43,44]. 

### 3.3. COVID-19 and Neonatal Complications

As far as the neonates are concerned, several complications have been reported. Giampiero Capobianco et al. stated a 39% complication rate among neonates born from infected women. In fact, fever, pneumonia and respiratory distress syndrome were the most frequent complications, indicating a possible outcome of the virus [43,44,45,46,47]. Other symptoms due to SARS-CoV-2 infection were also noticed. For example, tachycardia, thrombocytopenia, lymphocytopenia, leukocytosis, pneumothorax, vomiting, diarrhea, lethargy and septic shock [10,11,34,44]. Furthermore, there have been incidents of neonates, born from infected women, who attended the ICU. Daniele Di Mascio et al. and Kuma Diriba et al. observed that 8.7% and 11% of the newborns were admitted to the NICU, respectively, after facing fetal distress [3,42]. Similarly, J. Juan et al. and John Allotey et al. mentioned that approximately 33% of the neonates were transferred to the INCU, proving that the risk is higher when the mothers are infected with SARS-CoV-2.

### 3.4. COVID-19 and Vertical Transmission

Despite the high rate of cesarean sections, vaginal deliveries should be preferred when possible, taking into consideration that the majority of the results seem unable to prove the occurrence of a vertical transmission [3,8,14,33]. In fact, the analysis of biological samples usually finds no evidence of SARS-CoV-2 [9,15,24,45]. However, there have been cases which may indicate a possible transmission. Some neonates, delivered from infected mothers, soon after birth, have appeared positive for SARS-CoV-2 [3,5,10,24,25,41,44,46]. Giampiero Capobianco et al., in their study, stated that 6% of the neonates were infected by SARS-CoV-2 [7]. Furthermore, neonates have been presented with elevated IgM and IgG immediately after birth [9,11,14]. It is worth considering that IgM cannot pass the placenta, because of its large structure [9,14]. Instead, it is produced by the fetus itself after the infection. Additionally, IgMs need about 3 to 7 days after infection to increase [11,44]. The above arguments in combination may indicate a possible infection of the fetus while being in the uterus. Moreover, Ioannis Bellos et al., studied 17 cases which confirmed a perinatal transmission and noticed a correlation between transmission and hypothyroidism. This can be justified by the absence of thyroid hormones, affecting the development and function of the placenta [10]. All the above could potentially support a vertical transmission, but no certain conclusion can be made and more research is needed.

### 3.5. COVID-19 and Maternal–Fetal Monitoring

It is worth mentioning that COVID-19 does not negatively affect the majority of pregnancies. However, taking the above incidents and observations into consideration, it is obvious that there have been cases in which SARS-CoV-2 has badly influenced both the mother and the fetus/neonate. The virus can be associated with a dismal pregnancy outcome and, thus, pregnant women should be wisely supervised. Fetal monitoring by a daily non-stress test is advised for expectant mothers who are stable on standard oxygen therapy. Concerning patients in need of mechanical ventilation, ongoing monitoring is recommended after 28 weeks of pregnancy, so that early warning symptoms of an unsettling fetal condition may be identified [48,49].

### 3.6. COVID-19, Route and Timing of Delivery

To prevent fetal mortality and perhaps improve the mother’s cardiopulmonary function, a controlled birth (such as a cesarean section) is indicated if the respiratory condition is too crucial to handle, notably after 28 weeks of gestation. Several reasons are maternal dyspnea and hypoxia, fetal distress or concerns related to perinatal transmission [8,9,10,11,12]. Most pregnancies infected by SARS-CoV-2 were delivered with cesarean section. Giampiero Capobianco et al., when studying various publications, concluded that around 88% of women delivered this way [6,7,8,9]. The general consensus is that giving birth does not help pregnant women with acute respiratory failure recover faster, despite the fairly little research supporting this claim. The choice to proceed towards birth may be delayed in situations when the fetus is not developed enough to survive, particularly earlier than 24 weeks. Despite the lack of substantial evidence about the administration of corticosteroids for fetal lung maturity in the presence of SARS-CoV-2 infection, a single course of corticosteroids may be appropriate for individuals who are at a significant risk of preterm delivery within seven days [48,49]. 

### 3.7. COVID-19 Vaccination in Pregnancy

The vaccination of pregnant women reduces the increase in maternal and fetal morbidity related with COVID-19; consequently, all pregnant women should be vaccinated parallel with the rest of the population, depending on their age group and comorbidities. The Centers for Disease Control and Prevention (CDC), the Royal College of Obstetricians and Gynecologists (RCOG), the American College of Obstetricians and Gynecologists (ACOG), the Society for Maternal-Fetal Medicine, and the American College of Obstetricians and Gynecologists (ACOG) all recommend vaccination for all pregnant women [50,51].

Notably, three forms of COVID-19 vaccination are currently accessible globally: mRNA, viral vector, and inactivated. Vaccines employing mRNA particles (Pfizer or Moderna) are the most prevalent and have the greatest safety evidence. Antigen-presenting cells are responsible for mRNA uptake. These vaccines contain mRNA particles that induce muscle cells at the injection site to generate and activate a component of the SARS-CoV-2 spike protein [52].

In regions where mRNA vaccines are not easily accessible, viral vector and inactivated vaccines are also often administered to the general population; however, evidence on their safety in pregnant women is very limited. Notably, viral vector vaccines are not recommended for administration in pregnancy. Concerning timing of vaccination, the current research suggests that these immunizations are safe throughout pregnancy, even during the first trimester [53,54,55].

Despite the lack of randomized, pregnancy-specific evidence, there is absolutely no reason to expect that the efficacy of vaccinations would be compromised during pregnancy [56]. Immunogenicity studies reveal that mRNA vaccinations induce the same humoral immune response in pregnant and nonpregnant women [55,57]. Hence, mRNA vaccines are anticipated to provide the same degree of protection against SARS-CoV-2 infection and severe illness as those administered to nonpregnant patients.

Several longitudinal studies comparing the perinatal outcomes of vaccinated and unvaccinated pregnant women have showed good findings and no detrimental effects on pregnancy or the newborn. Vaccination with mRNA vaccines does not increase the risk of spontaneous abortion, premature delivery, low birthweight, maternal or neonatal intensive care unit admission, fetal mortality, congenital abnormalities, or pulmonary embolism [58].

Notably, the literature reports rare instances of mRNA vaccine-associated myocarditis, as well as vaccine-induced thrombosis and thrombocytopenia; however, evidence is still scarce [59,60].

Another concern is the necessity for pregnant women to receive booster doses, since the effectiveness of immunization diminishes with time [32]. Women who received inactivated or viral vector immunizations may benefit from a booster injection. Booster vaccinations are advised at least two months following a first immunization using viral vector vaccines; the booster dose should consist of an mRNA vaccine [51]. 

In conclusion, observational studies corroborate the findings of randomized trials that mRNA immunization is extremely effective in preventing severe SARS-CoV-2 infection in pregnant women, emphasizing that the potential maternal and fetal benefits of vaccination significantly outweigh the potential risks. To guarantee that pregnant women have unrestricted access to the COVID-19 vaccine should be a global priority.

### 3.8. COVID-19 and Pharmacological Treatment during Pregnancy

Pharmacological interventions in expectant women with COVID-19 should primarily aim to reduce fetal hazards. Data on the administration of medication for SARS-CoV-2-infected pregnant women are still sparse. Anticoagulants, anti-inflammatory/immunomodulatory medicines, and off-label antivirals are the pharmacological classes that are utilized. Notably, prior to any medication, oxygen should be provided to maintain saturation at 94–98%, and the hourly fluid input/output should be recorded.

The use of aspirin may be helpful in cases of severe COVID-19 unless there is an increased risk of bleeding incidents or the platelet count is below 50 × 10^9^/L [20]. NSAIDs (Non-steroidal anti-inflammatory drugs), such as ibuprofen, have been demonstrated to result in adverse effects on the growing fetus after 20 weeks of gestation, and thus should not be used to alleviate mild COVID-19 symptoms in this population [61]. 

The most frequently used anticoagulant during pregnancy is low-molecular-weight heparin (LMWH), given its effectiveness and safety in several prospective clinical studies. Heparin should be used as a preventative measure for all pregnant patients who are being hospitalized due to SARS-CoV-2 infection and prescribed for at least ten days after discharge [56,62]. 

Corticosteroids have been demonstrated to be effective and are currently advised for use in pregnant COVID-19 patients who require mechanical ventilation or additional oxygen, with caution concerning their prolonged administration. Dexamethasone 12 mg twice (24 h apart) followed by oral prednisolone 40 mg once daily or intravenous hydrocortisone 80 mg twice daily is the optimal course of treatment for fetal lung maturation. If maturation of the fetal lungs is not necessary, oral prednisolone 40 mg once daily or intravenous hydrocortisone 80 mg twice daily should be provided [20,63]. 

Several antiviral medications are restricted during pregnancy due to their known teratogenic effects. There are pharmacologic medications that have a lower risk of teratogenicity than others that can be used to swiftly and successfully treat SARS-CoV-2 infection during pregnancy. Remdesivir, an intravenous antiviral with high effectiveness against SARS-CoV-2, acts by preventing RNA-dependent RNA polymerase from replicating RNA, which inhibits viral growth. This medication has been administered to pregnant patients receiving oxygen therapy, whether intubated or not, without causing fetal harm. Remdesivir should not be administered during pregnancy unless the patient’s clinical status warrants it, although the literature on the efficacy of remdesivir is always changing [64,65]. Tocilizumab, a humanized monoclonal antibody against the IL-6 receptor has not been correlated with teratogenic outcomes and should be considered in patients with hypoxia and signs of systemic inflammation, since it may improve the outcome [20,66]. 

It is debatable if colchicine should be used during pregnancy. According to certain data, colchicine medication did not significantly raise the risk of fetal deformity or abortion during pregnancy [67,68,69]. Orally administered molnupiravir is an antiviral prodrug that prevents viral replication, although its safety during pregnancy has not yet been studied in humans. As a result, molnupiravir is not advised for use during pregnancy until more research has been conducted [70,71,72]. 

The use of antibiotics is not recommended in viral infections including COVID-19; however, some regimens do suggest them in cases of bacterial pneumonia, with azithromycin, a macrolide antibiotic being the most commonly used therapeutic agent [54]. The suggested regimen contains 250 or 500 mg of azithromycin once daily, for up to 5 days, either intravenously or orally administered [73,74]. 

Paxlovid, a second antiretroviral oral medicine available to treat individuals who are at risk of progressing to severe COVID-19, has not been studied in pregnant women, so its use is not yet recommended [75]. Finally, it is worth highlighting that the aforementioned treatment options present a low risk of pharmacological interaction with already established interventions for other pregnancy-related conditions, such as hypertensive disorders in pregnancy, and thus can be safely co-administered [76].

## 4. Conclusions

Pregnant women’s infection with SARS-CoV-2 has been correlated with unfavorable maternal and newborn outcomes. Pneumonia, ICU hospitalization, mechanical ventilation, as well as a possibly higher mortality risk have been reported. The chances of pre-eclampsia, stillbirth, and premature delivery were also elevated. Cases of miscarriages and perinatal deaths have been stated, as well as some stillbirths and intrauterine growth restriction. Regarding the method of delivery, most pregnancies infected by SARS-CoV-2 were delivered with cesarean section, although the motives behind this decision are yet unclear. Despite the high rate of cesarean sections, vaginal deliveries should be preferred when possible. As far as the neonates are concerned, several complications have been reported including fever, pneumonia, and respiratory distress syndrome. Furthermore, there have been incidents of neonates born from infected women who attended the ICU. In order to avoid the previously mentioned adverse effects, thorough monitoring based both on the severity of maternal infection and gestational age should be implemented. 

According to RCOG, although there is an increased risk of severe disease during the third trimester, the danger of dying is still quite low. Risk factors correlated with both COVID-19 infection and hospitalization are being unvaccinated, co-morbidities such as diabetes and hypertension, Black or Asian race, increased maternal age and body mass index, socioeconomic deprivation, and health or public-related occupations. The course of the infection may differ depending on the specific SARS-CoV-2 variant. Particularly, the Delta variant is linked to more severe disease than the Alpha and Omicron versions. If necessary, chest imaging should be obtained in COVID-19-symptomatic women. If clinical signs of deterioration are present, such as decreased urine output, acute renal injury, sleepiness, increased demands for oxygen, and tachypnea, this should immediately prompt a reassessment of the patient’s treatment. Considerations must be taken concerning whether emergency cesarean birth or labor induction is needed in cases of serious fetal distress or in order to enhance maternal resuscitation. 

There is an increased rate of preterm birth, especially iatrogenic, in infected patients. Furthermore, an association between maternal COVID-19 and an increased risk of stillbirth and small-for-gestational-age infants has been recorded. Vertical transmission rarely occurs, but the incidence of congenital anomalies does not appear to increase. The prenatal care should be provided as usual wherever practicable. Evaluation of the fetal biometry should be performed within the first 14 days after recovery in women with serious illness. 

All pregnant women should be vaccinated against COVID-19, obtaining two doses and a booster, since it reduces the risk of hospitalization and ICU admission. The preferred vaccines are Pfizer-BioNTech or Moderna and they can be administered at any point during pregnancy or lactation. COVID-19-symptomatic women should be received continuous electronic feta monitoring during labor in an obstetric care unit. 

Future research is required to gather more reliable data to further confirm or elucidate these findings, comprehend the pathophysiologic processes that account for these relationships, and establish practical preventative measures for SARS-CoV-2-positive pregnant women.
